# High prevalence of mental disorders: a population-based cross-sectional study in the city of Ilam, Iran

**DOI:** 10.3389/fpsyt.2023.1166692

**Published:** 2023-06-13

**Authors:** Hojatollah Kakaei, Farajolah Maleki, Azam Biderafsh, Reza Valizadeh, Mohammad Ali Mansournia, Iraj Pakzad, Reza Pakzad

**Affiliations:** ^1^Department of Occupational Health, Faculty of Health, Ilam University of Medical Sciences, Ilam, Iran; ^2^Non-Communicable Diseases Research Center, Ilam University of Medical Sciences, Ilam, Iran; ^3^Department of Epidemiology, School of Public Health, Tehran University of Medical Sciences, Tehran, Iran; ^4^Psychosocial Injuries Research Center, Ilam University of Medical Sciences, Ilam, Iran; ^5^Department of Microbiology, School of Medicine, Ilam University of Medical Sciences, Ilam, Iran; ^6^Department of Epidemiology, Faculty of Health, Ilam University of Medical Sciences, Ilam, Iran; ^7^Student Research Committee, Ilam University of Medical Sciences, Ilam, Iran

**Keywords:** prevalence, cross-sectional study, mental disorders, depression, anxiety, stress

## Abstract

**Aim:**

To determine the age- and sex-standardized prevalence and risk factors of depression, anxiety, and stress symptoms in the city of Ilam.

**Method:**

In this population-based cross-sectional study, 1,350 people were invited using a multi-stage stratified cluster random sampling method. Depression, anxiety, and stress symptoms were measured using the DASS-21 standard questionnaire. For data analysis, multiple ordinal logistic regression was used in Stata version 12 software. A significance level of 5% was considered.

**Results:**

The data of 1,431 people were analyzed. The age- and sex-standardized prevalence (95% CI) of severe depression, anxiety, and stress symptoms was 19.90% (17.64 to 22.16), 25.95% (23.48 to 28.43), and 15.75% (13.69 to 17.81), respectively. There was a positive association among depression symptoms with female sex (OR: 1.52; *p* < 0.003), Kurdish ethnicity (OR: 2.15; *p* < 0.004), low educational level (OR: 1.37; *p* < 0.031), job losing history (OR: 1.64; *p* < 0.001), mental disorders history (OR: 2.17; *p* < 0.001), hopelessness for the future (OR: 5.38; *p* < 0.001), and history of other diseases (OR: 1.67; *p* < 0.001). There was a positive association among anxiety symptoms with female sex (OR: 1.72; *p* < 0.001), job losing history (OR: 1.53; *p* < 0.003), mental disorders history (OR: 2.11; *p* < 0.001), hopelessness to future (OR: 3.33; *p* < 0.001) and history of other diseases (OR: 1.97; *p* < 0.001). Hopelessness for the future and a history of other diseases were the most effective variables for anxiety symptoms and stress symptoms.

**Conclusion:**

A significant proportion of Ilam’s urban population suffers from mental disorders. Increasing people’s awareness, establishing counseling centers, and improving infrastructure should be considered by mental health policymakers who work in the province.

## 1. Introduction

Mental disorders constitute a major cause of disability across the world (1). Of the various indicators of mental disorders, depression, anxiety, and stress are the most prominent (2) with depression being the most prevalent and responsible for a significant proportion of the burden of mental illness globally (3, 4). According to the World Health Organization, an estimated 5% of adults worldwide suffer from depression, and one in four individuals experience a mental health condition at some point during their lifetime (5). Recent research suggests that the global prevalence of mental disorders ranges between 11% and 23.8%, with adults being affected at a rate of 12.6% to 48.6% (6, 7). Furthermore, the one-year prevalence of mental disorders varies between 3.4% and 26.4%, with the Eastern Mediterranean regions exhibiting the highest prevalence rates of 15.6% to 35.5% (3).

The prevalence of mental disorders in Iran is consistent with the global trend, with depression and anxiety being the most commonly diagnosed conditions (8, 9) and their burden on the population is also increasing (10). Studies indicate that approximately 7 million people in Iran are affected by some form of mental disorder, with approximately 15%–20% of the population experiencing symptoms of depression ranging from mild to severe. These trends are attributed to heightened stress levels caused by social and environmental changes, as well as a rise in specific physical ailments (11, 12).

The results of a study in Iran showed that 31.03% of the population is affected by mental disorders (13). The survey by Hajebi et al. showed the prevalence of anxiety in people 15–64 years. Old was 15.6% (14). Another study with a substantial sample size reports that 11% of Iranians have some form of mental disorder (9). Najafipour et al. (15) and Mohammadi et al. (16) showed that 25.4 and 59.5% of residents in southeast Iran suffered from anxiety and depression, respectively. Further studies in Kashan (17) and Iran (18, 19), found the prevalence of mental disorders to be between 23%–39.6%. However, there have been few studies of residents of western Iran. According to Shirzadi et al. (20), the prevalence of depression and anxiety disorders in Kermanshah was 8.3% and 4.7%, respectively. Finally, according to Veisani et al. (21), 26.1% of Ilamian people had one or more mental disorders.

Mental disorders can occur in complex interactions between social, psychological, and biological factors. The risk of developing mental disorders increases with poverty, unemployment, childhood adversity, life events such as the death of family members, physical illness, and alcohol and drug abuse (22). Previous research has identified several key variables that impact mental health, including age, gender, income, access to healthcare services, physical and social environment, education and literacy levels, personal hygiene habits, and coping skills (23–25).

Due to its distinct geographical features, poor welfare and development infrastructure, high unemployment rate, and the effects of the imposed conflict, Ilam province has a greater frequency of mental disorders (23). As a result, the suicide rate in Ilam is higher than the average for the world and has been rising recently (26). Mental disorders lead to a decrease in one’s quality of life and have detrimental effects on human health, performance, and efficiency, and their impacts can be long-term and affect a person’s ability to function and live a healthy life. Therefore, it is necessary to pay special attention to the mental health state of the community. The need for comprehensive information about mental health in order to plan and make policies led us to conduct a study with the aim of determining the prevalence of stress, anxiety and depression in the population of Ilam.

## 2. Methods

### 2.1. Study population

**Figure 1:**
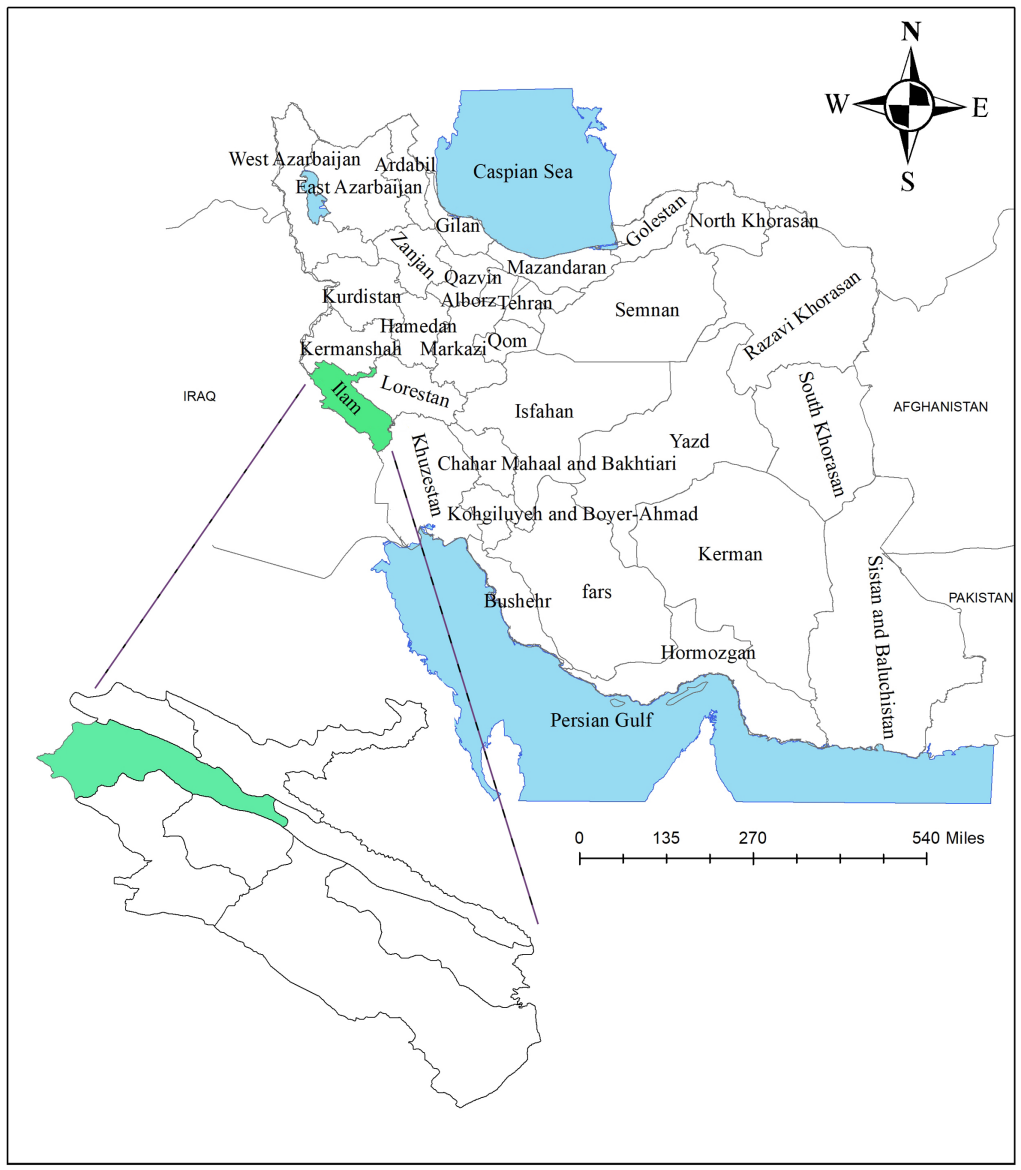
Geographical location of Ilam city.

All parts of the present study are based on the Strengthening the Reporting of Observational Studies in Epidemiology (STROBE) checklist. This was a population-based cross-sectional study that was conducted on all the citizens of the city of Ilam. The city of Ilam, located in the west of Iran with a population of 200,931 people, is known as the least populated center of the country’s province. The location of Ilam on the map is shown in Figure 1.

According to the study by Montazeri et al. (27), the prevalence of depression in Iran ranges from 6% to 73%. Considering the prevalence of depression being equal to 30%, a confidence level of 95%, and the precision = 1/10 of the prevalence, based on the following formula, a minimum sample size of 896 was estimated.

$$n=\frac{z^{2}\;pq}{d^{2}}=\frac{1.96^{2}*0.3*0.7}{0.03^{2}}=896$$

According to the type of sampling, considering the design effect equal to 1.5 sample size, 1,344 people were estimated, and finally, 1,350 people were considered as the minimum final sample size (28). Multi-stage stratified cluster random sampling with a proportion-to-size approach was used to select the participants. For this purpose, the city of Ilam was divided into 10 regions based on 10 community health centers, and the population covered by each region was obtained. Then, according to the population of each region, the proportion of the sample size that should be selected from each region was determined. After determining the sample size in each region, cluster sampling was implemented.

The sample size in each cluster was considered to be 20 people so the clustered map was prepared separately for each region and the clusters were selected according to the sample size in each center. After identifying each cluster, the sampling team was placed on the southwest side of this cluster by referring to the address of the cluster. The team then started to sample the households counter-clockwise and this process continued until the sample size of each cluster was exhausted. It should be noted that all family members over 15 years of age were included in the study.

After explaining the objectives of the study and obtaining informed consent, the sample team collected the study information by questionnaire. This questionnaire has two parts. The first part includes demographic information (age, gender, race, education, household dimensions, occupation, and insurance coverage), economic status (which included 15 questions about assets and was created using the principle component analysis method of economic variables), and other information (co-morbidities, history of mental disorder, history of death of family members, history of job loss and one’s hope for the future).

The second part included the Depression-Anxiety-Stress-Scales (DASS) questionnaire (21-question version), which includes three domains and each domain has seven questions. The scoring of each question was on a Likert scale (0 to 3) and the final score of each was obtained through the sum of the scores of the related questions. The validity and reliability of this tool were previously confirmed by Sahebi et al. (29) (Cronbach’s alpha for depression, anxiety, and stress were 77, 79, and 78%, respectively).

### 2.2. Statistical analysis

To estimate the age- and sex-standardized prevalence, the sample taken was weighted based on the Ilam city population. Then, the age- and sex-standardized prevalence of depression, anxiety, and stress was estimated with a 95% confidence interval. First, the chi-squared test and *t*-test were used to check the association between the studied outcomes and demographic variables. Then, multiple ordinal logistic regression was used for model building and checking the simultaneous effect of the study variables on the outcomes.

It should be noted that the criteria for entering the variables into the multiple ordinal logistic regression model was a significance level of less than 0.2, and the cluster effect was considered in order to correct the sampling error. The standardized coefficients were used to determine the most important effective variables in the model. All analyses were performed by using Stata version 12 and a significance level of 0.05 was considered.

## 3. Results

The data of 1,431 participants were analyzed. Table 1 shows the distribution of demographic and other variables in the present study. The mean age (SD) of the participants was 40.43 (15.51), 50.38% (47.79 to 52.98) of the participants were female, 94.34% (93.14 to 95.54) were of Kurdish ethnicity, 61.29% (58.76 to 63.81) were married, 35.64% (33.16 to 38.12) had an education level of more than a bachelor’s degree, and 30.75% (28.35 to 33.14) were classified as a high economic group. In total, 8.03% (6.62 to 9.45) of the participants had a history of mental disorder and just 57.02% (54.45 to 59.59) of participants had hope for the future.

**Table 1:** Distribution of demographic variables in study population.

Variable		Number	Percent (95% CI)
Sex	Male	710	49.62% (47.02 to 52.21)
	Female	721	50.38% (47.79 to 52.98)
Race	Kurd	1,350	94.34% (93.14 to 95.54)
	Other	81	5.66% (4.46 to 6.86)
Marital status	Married	877	61.29% (58.76 to 63.81)
	Single	474	33.12% (30.68 to 35.57)
	Divorce or widow	80	5.59% (4.41 to 6.78)
Education	<Diploma	447	31.24% (28.83 to 33.64)
	Diploma and associate degree	474	33.12% (30.68 to 35.57)
	≥Bachelor	510	35.64% (33.16 to 38.12)
Job	Student	206	14.40% (12.57 to 16.22)
	Employed	493	34.45% (31.99 to 36.92)
	Retrieved	155	10.83% (9.22 to 12.44)
	Housekeeper or unemployed	577	40.32% (37.78 to 42.87)
Insurance	Cover	1,084	75.75% (73.53 to 77.97)
	Un-covered	347	24.25% (22.03 to 26.47)
Economic statue	Low	545	38.09% (35.57 to 40.61)
	Middle	446	31.17% (28.76 to 33.57)
	High	440	30.75% (28.35 to 33.14)
Depression^b^	No	668	53.07% (50.24 to 55.88)
	Moderate	357	27.03% (24.52 to 29.54)
	Severe	275	19.90% (17.64 to 22.16)
Anxiety^b^	No	684	54.14% (51.33 to 56.96)
	Moderate	274	19.90% (17.64 to 22.16)
	Severe	358	25.95% (23.48 to 28.43)
Stress^b^	No	806	64.10% (61.38 to 66.81)
	Moderate	266	20.15% (17.88 to 22.42)
	Severe	206	15.75% (13.69 to 17.81)
Loss of family members (yes)		674	47.10% (44.51 to 49.69)
Job loss history (yes)		286	19.98% (17.91 to 22.06)
Mental disorder history (yes)		115	8.03% (6.62 to 9.45)
Hope for the future (yes)		816	57.02% (54.45 to 59.59)
History of other diseases (yes)		503	35.15% (32.67 to 37.63)
^a^Age (years old)		1,431	40.43 ± 15.51
^a^BMI (kg/m^2^)		1,431	25.77 ± 4.18

a Present as mean ± SD.

b Age—sex standardized.

As shown in Table 1, the age- and sex-standardized prevalence of severe depression, anxiety, and stress symptoms was 19.90% (17.64 to 22.16), 25.95% (23.48 to 28.43), and 15.75% (13.69 to 17.81), respectively. The age-sex standardized prevalence of moderate depression, anxiety, and stress symptoms was 27.03% (24.52 to 29.54), 19.90% (17.64 to 22.16), and 20.15% (17.88 to 22.42), respectively.

Table 2 shows the association between the mental disorders and different variables. As shown in Table 2, the prevalence of severe depression symptoms in females (25%) was significantly more than in males (17.33%) (*p* < 0.003). This pattern was observed for anxiety symptoms (31.15% vs. 23.30%; *p* < 0.001) and stress symptoms (18.64% vs. 13.64%; *p* < 0.012). Furthermore, those of Kurdish ethnicity were more likely to have severe depression symptoms (*p* < 0.045) or anxiety symptoms (*p* < 0.049). The participants who had educational levels lower than a diploma were more likely to have severe depression (*p* < 0.003), anxiety (*p* < 0.016), and stress symptoms (*p* < 0.023). Other variables including working as a housekeeper or being unemployed (28.32%; *p* < 0.001), lack of insurance coverage (27.60% vs. 19.15%; *p* < 0.006), low economic status (29.28%; *p* < 0.001); history of job losing (28.68% vs. 19.29%; *p* < 0.001); history of mental disorders (43.12% vs. 19.14%; *p* < 0.001); hopelessness for the future (39.63% vs. 8.24%; *p* < 0.001), and history of other diseases (30.16% vs. 16.53%; *p* < 0.001) had a positive effect on the severity of depression symptoms. Age, BMI, and marital status had no association with depression symptoms.

**Table 2:** Association between the mental disorders with different variables based on the chi-squared test.

Variables		Depression *N* (%)				Anxiety *N* (%)				Stress *N* (%)			
		No	Moderate	Severe	*p*-value	No	Moderate	Severe	*p*-value	No	Moderate	Severe	*p*-value
Sex	Male	335 (54.45)	184 (28.22)	113 (17.33)	0.003	385 (58.25)	122 (18.46)	154 (23.30)	<0.001	431 (66.82)	126 (19.53)	88 (13.64)	0.012
	Female	313 (48.30)	173 (26.70)	162 (25.00)		299 (45.65)	152 (23.21)	204 (31.15)		375 (59.24)	140 (22.12)	118 (18.64)	
Ethnicity	Kurd	618 (50.53)	343 (28.05)	262 (21.42)	0.045	633 (51.13)	263 (21.24)	342 (27.63)	0.049	750 (62.45)	252 (20.98)	199 (16.57)	0.136
	Other	50 (64.94)	14 (18.18)	13 (16.88)		65 (65.98)	11 (14.10)	16 (20.51)		56 (72.73)	14 (18.18)	7 (9.09)	
Marital status	Married	417 (52.72)	221 (27.94)	153 (19.34)	0.285	423 (53.01)	166 (20.80)	209 (26.19)	0.224	497 (63.96)	170 (21.88)	110 (14.16)	0.148
	Single	217 (49.89)	117 (26.90)	101 (23.22)		231 (52.26)	89 (20.14)	122 (27.60)		267 (62.38)	81 (18.93)	80 (18.69)	
	Divorce /widow	34 (45.95)	19 (25.68)	21 (28.38)		30 (39.47)	19 (25.00)	27 (35.53)		42 (57.53)	15 (20.55)	16 (21.92)	
Education	<Diploma	208 (51.49)	96 (23.76)	100 (24.75)	0.003	197 (48.52)	79 (19.46)	130 (32.02)	0.016	245 (62.03)	70 (17.72)	80 (20.25)	0.023
	Diploma and associate degree	205 (47.90)	121 (28.27)	102 (23.83)		222 (50.80)	90 (20.59)	125 (28.60)		261 (62.59)	88 (21.1)	68 (16.31)	
	≥Bachelor	255 (54.49)	140 (29.91)	73 (15.60)		265 (56.03)	105 (22.20)	103 (21.78)		300 (64.38)	108 (23.18)	58 (12.45)	
Job	Student	106 (55.50)	52 (27.23)	33 (17.28)	<0.001	98 (50.26)	54 (27.69)	43 (22.05)	<0.001	119 (62.96)	45 (23.81)	25 (13.23)	<0.001
	Employed	258 (55.60)	121 (26.08)	85 (18.32)		265 (57.73)	84 (18.30)	110 (23.97)		312 (68.12)	92 (20.09)	54 (11.79)	
	Retrieved	79 (56.43)	47 (33.57)	14 (10.01)		88 (60.69)	24 (16.55)	33 (22.76)		96 (70.59)	25 (18.38)	15 (11.03)	
	Housekeeper or unemployed	225 (44.55)	137 (27.13)	143 (28.32)		233 (45.07)	112 (21.66)	172 (33.27)		279 (56.36)	104 (21.01)	112 (22.63)	
Insurance	Cover	526 (53.02)	276 (27.82)	190 (19.15)	0.006	532 (52.99)	212 (21.12)	260 (25.90)	0.159	628 (64.61)	197 (20.27)	147 (15.12)	0.100
	Un-covered	142 (46.10)	81 (26.30)	85 (27.60)		152 (48.72)	62 (19.87)	98 (31.41)		178 (58.17)	69 (22.55)	59 (19.28)	
Economic statue	Low	200 (41.24)	143 (29.48)	142 (29.28)	<0.001	220 (45.08)	94 (19.26)	174 (35.66)	<0.001	266 (56.72)	104 (22.17)	99 (21.11)	0.001
	Middle	234 (57.21)	108 (26.41)	67 (16.38)		238 (57.21)	90 (21.63)	88 (21.15)		275 (68.07)	80 (19.8)	49 (12.13)	
	High	234 (57.64)	106 (26.11)	66 (16.26)		226 (54.85)	90 (21.84)	96 (23.30)		265 (65.43)	82 (20.25)	58 (14.32)	
Loss of family members	No	361 (53.17)	190 (27.98)	128 (18.85)	0.101	365 (53.21)	154 (22.45)	167 (24.34)	0.038	439 (64.75)	142 (20.94)	97 (14.31)	0.165
	Yes	307 (49.44)	167 (26.89)	147 (23.67)		319 (50.63)	120 (19.05)	191 (30.32)		367 (61.17)	124 (20.67)	109 (18.17)	
Job loss history	No	563 (54.03)	278 (26.68)	201 (19.29)	<0.001	571 (54.33)	210 (19.98)	270 (25.69)	0.003	663 (64.68)	216 (21.07)	146 (14.24)	0.001
	Yes	105 (40.70)	79 (30.62)	74 (28.68)		113 (42.64)	64 (24.15)	88 (33.21)		143 (56.52)	50 (19.76)	60 (23.72)	
Mental disorder history	No	642 (53.90)	321 (26.95)	228 (19.14)	<0.001	654 (54.23)	251 (20.81)	301 (24.96)	<0.001	766 (65.14)	240 (20.41)	170 (14.46)	<0.001
	Yes	26 (23.85)	36 (33.03)	47 (43.12)		30 (27.27)	23 (20.91)	57 (51.82)		40 (39.22)	26 (25.49)	36 (35.29)	
Hope for the future	No	151 (28.22)	172 (32.15)	212 (39.63)	<0.001	183 (34.08)	122 (22.72)	232 (43.20)	<0.001	245 (46.85)	132 (25.24)	146 (27.92)	<0.001
	Yes	517 (67.58)	185 (24.18)	63 (8.24)		501 (64.31)	152 (19.51)	126 (16.17)		561 (74.3)	134 (17.75)	60 (7.95)	
History of other diseases	No	494 (57.51)	223 (25.96)	142 (16.53)	<0.001	505 (58.38)	190 (21.97)	170 (19.65)	<0.001	582 (68.79)	161 (19.03)	103 (12.17)	<0.001
	Yes	174 (39.46)	134 (30.39)	133 (30.16)		179 (39.69)	84 (18.63)	188 (41.69)		224 (51.85)	105 (24.31)	103 (23.84)	
^*^Age		40.32 ± 15.73	39.82 ± 15.42	40.68 ± 14.80	0.778	39.99 ± 15.46	38.97 ± 15.26	41.48 ± 15.64	0.116	40.23 ± 15.52	39.39 ± 14.94	40.43 ± 15.44	0.699
^*^BMI		25.55 ± 3.92	25.79 ± 4.15	26.27 ± 4.91	0.062	25.57 ± 3.88	25.73 ± 4.26	26.09 ± 4.73	0.165	25.61 ± 3.95	25.96 ± 4.15	25.99 ± 5.03	0.310

The association of mentioned variables with anxiety symptoms was also the same, so that people who experienced job loss (33.21% vs. 25.69%; *p* < 0.001), loss of family members (30.32% vs. 24.34%; *p* < 0.038), mental disorder history (51.82% vs. 24.96%; *p* < 0.001), hopelessness to future (43.20% vs. 16.17%; *p* < 0.001) and history of other diseases (41.69% vs. 19.65%; *p* < 0.001), had a high level of anxiety symptoms. The association between stress symptoms with demographic variables also is shown in Table 2.

Table 3 showed the association between mental disorders with study variables using a multiple ordinal logistic regression model. As shown in Table 3, there was a positive association between female sex and depression symptoms (OR with 95% CI: 1.52; 1.16 to 1.98), anxiety symptoms (OR with 95% CI: 1.72; 1.32 to 2.25), and stress symptoms (OR with 95% CI: 1.71; 1.31 to 2.22), adjusted for other variables. Furthermore, the probability of depression symptoms (OR with 95% CI: 2.15; 1.28 to 3.61), and anxiety symptoms (OR with 95% CI: 2.08; 1.26 to 3.44) in those of Kurdish ethnicity was more than other ethnicities. Although education level had a significant association with anxiety symptoms and stress symptoms in the simple analysis, this association was not observed in the multiple analysis. The association between education and depression symptoms remained significant as the probability of depression symptoms was higher in participants with diplomas and associate degrees than in those with a lower diploma (OR with 95% CI: 1.37; 1.03 to 1.83).

**Table 3:** Association between the mental disorders with different variables multiple ordinal logistic regression.

Variables		Depression		Anxiety		Stress	
		OR (95% CI)	*p*-value	OR (95% CI)	*p*-value	OR (95% CI)	*p*-value
Sex	Male	**Reference**	**—**	**Reference**	**—**	**Reference**	**—**
	Female	1.52 (1.16 to 1.98)	0.003^a^	1.72 (1.32 to 2.25)	<0.001^a^	1.71 (1.31 to 2.22)	<0.001^a^
Ethnicity	Other	**Reference**	**—**	**Reference**	**—**	Not included	**—**
	Kurd	2.15 (1.28 to 3.61)	0.004^a^	2.08 (1.26 to 3.44)	0.004^a^		
Education	<Diploma	**Reference**	**—**	**Reference**	**—**	**Reference**	**—**
	Diploma and associate degree	1.37 (1.03 to 1.83)	0.031^a^	1.09 (0.82 to 1.45)	0.539	1.09 (0.83 to 1.45)	0.533
	≥Bachelor	1.14 (0.84 to 1.54)	0.407	0.98 (0.72 to 1.32)	0.880	0.98 (0.73 to 1.32)	0.902
Job	Student	**Reference**	**—**	**Reference**	**—**	**Reference**	**—**
	Employed	0.87 (0.6 to 1.26)	0.458	0.74 (0.51 to 1.07)	0.115	0.75 (0.53 to 1.07)	0.116
	Retrieved	0.61 (0.38 to 0.99)	0.046^a^	0.54 (0.33 to 0.9)	0.017^a^	0.56 (0.35 to 0.89)	0.015^a^
	Housekeeper or unemployed	0.96 (0.67 to 1.38)	0.844	0.79 (0.55 to 1.13)	0.195	0.81 (0.57 to 1.14)	0.231
Insurance	Cover	**Reference**	**—**	Not included	**—**	Not included	**—**
	Un-covered	1.14 (0.86 to 1.5)	0.359				
Economic status	Low	**Reference**	**—**	**Reference**	**—**	**Reference**	**—**
	Middle	0.64 (0.49 to 0.85)	0.002^a^	0.70 (0.53 to 0.92)	0.010^a^	0.71 (0.54 to 0.94)	0.015^a^
	High	0.7 (0.52 to 0.93)	0.015^a^	0.85 (0.64 to 1.14)	0.278	0.88 (0.66 to 1.17)	0.382
Job loss history	No	**Reference**	**—**	**Reference**	**—**	Not included	**—**
	Yes	1.64 (1.24 to 2.18)	0.001^a^	1.53 (1.16 to 2.02)	0.003^a^		
Mental disorder history	No	**Reference**	**—**	**Reference**	**—**	**Reference**	**—**
	Yes	2.17 (1.47 to 3.21)	<0.001^a^	2.11 (1.42 to 3.14)	<0.001^a^	1.51 (1.14 to 1.99)	0.004^a^
Hope for the future	Yes	**Reference**	**—**	**Reference**	**—**	**Reference**	**—**
	No	5.38 (4.25 to 6.81)	<0.001^a^	3.33 (2.65 to 4.18)	<0.001^a^	2.17 (1.46 to 3.22)	<0.001^a^
History of other diseases	No	**Reference**	**—**	**Reference**	**—**	**Reference**	**—**
	Yes	1.67 (1.3 to 2.15)	<0.001^a^	1.97 (1.54 to 2.53)	<0.001^a^	3.32 (2.65 to 4.16)	<0.001^a^
Loss of family members	No	Not included	**—**	**Reference**	**—**	**Reference**	**—**
	Yes			1.05 (0.83 to 1.34)	0.683	1.97 (1.53 to 2.52)	<0.001^a^

a Significant at 0.05 level.

Moreover, the result of the analysis showed that higher economic status had a negative association with depression symptoms as the probability of having moderate or severe depression symptoms was 0.7 (95% CI: 0.52 to 0.93) in participants with high economic status, in comparison to those with a lower economic status. This pattern for anxiety symptoms and stress symptoms was shown only for middle economic status. In other words, in participants with middle economic status, the probability of having moderate or severe anxiety and stress symptoms was 0.70 (95% CI: 0.53 to 0.92) and 0.71 (95% CI: 0.54 to 0.94), respectively, in comparison to lower economic status.

Our analysis also showed that a history of losing a job (OR with 95% CI: 1.64; 1.24 to 2.18), history of mental disorders (OR with 95% CI: 2.17; 1.47 to 3.21), hopelessness for the future (OR with 95% CI: 5.38; 4.25 to 6.81), and history of other diseases (OR with 95% CI: 1.67; 1.30 to 2.15) lead to increases in the probability of depression symptoms. A similar pattern was seen for anxiety symptoms. Seen together, job loss history (OR with 95% CI: 1.53; 1.16 to 2.02), mental disorders history (OR with 95% CI: 2.11; 1.42 to 3.14), hopelessness for the future (OR with 95% CI: 3.33; 2.65 to 4.18) and history of other diseases (OR with 95% CI: 1.97; 1.54 to 2.53) had a positive association with anxiety symptom levels. For stress symptoms, in addition to the mentioned variables, the loss of family members was also associated with an increased probability of stress symptoms (OR with 95% CI: 1.97; 1.53 to 2.52).

The standardized coefficient was estimated to determine the most effective variables in each model. As shown in Table 4, the most effective variables for depression symptoms were hopelessness for the future (−0.668) and a history of other diseases (0.668). Furthermore, the most effective variable for anxiety symptoms and stress symptoms was a history of other diseases (0.745 and 0.765, respectively).

**Table 4:** Standardized coefficients to determine the most effective variables on the outcomes.

Variables	Standardized coefficient		
	Depression	Anxiety	Stress
Sex	0.236	0.287	0.268
Ethnicity	−0.126	−0.136	Not included
Education	0.040	−0.039	−0.024
Job	−0.028	−0.072	−0.064
Insurance	0.069	Not included	Not included
Economic status	−0.136	−0.048	−0.049
Job loss history	0.158	0.105	Not included
Mental disorder history	0.222	0.216	0.229
Hope for the future	−0.668	−0.403	−0.396
History of other diseases	0.668	0.745	0.765
Loss of family members	Not included	−0.039	−0.042

## 4. Discussion

According to the results, less than 50% of the study population have experienced different levels of depression, anxiety, and stress symptoms. The prevalence of mental disorders estimated in this study is consistent with the findings of previous national mental health surveys conducted in Iran in 2001, 2002, and 2012, which reported the occurrence of mental disorders to be 17, 21, and 26.3%, respectively.

It should be noted that due to the use of various measurement tools, population groups, and diagnostic techniques, the prevalence of mental disorders may range significantly between various geographic regions. The results of this study were, however, almost identical to those of the national mental health surveys, and the small variation in the estimated prevalence can be attributed to the varied statistical population and various estimation methods (9, 19, 26). Moreover, a meta-analysis of 46 research studies found that depression was prevalent in 34.26% of people, which is comparable with the findings of the present study (30).

The current study found that the prevalence of depression, anxiety, and stress symptoms was significantly higher in women compared to men, with the prevalence of depression at 25% and 17.3%, anxiety at 31.15% and 23.3%, and stress at 18.6% and 13.6%, respectively. This finding is consistent with previous research conducted globally (31–33) and in Iran (34–36), which has suggested that women have a higher prevalence of mental disorders due to various factors such as social limitations, environmental stress, physiological factors, and hormonal differences (37–39).

As would be expected, there is an association between education level and stress, anxiety, and depression symptoms, such that higher rates of these disorders were associated with lower levels of education. This is consistent with other studies (26, 40). Higher levels of education are associated with better communication skills, access to helpful resources, and a more favorable social and economic situation that enable people to manage life’s challenges. People with lower levels of education have a higher prevalence of depression due to socio-cultural limitations and the inability to manage these emotions effectively (10, 35). Our findings, however, were not consistent with Chegini’s et al. study (41), which may be explained by the fact that they also considered paranoid and mood disorders in addition to stress, anxiety, and depression (41).

There is an association between Kurdish ethnicity and lower levels of depression symptoms and anxiety symptoms. It appears that in societies with diverse ethnicities, the majority ethnicity has greater social support, leading to better coping mechanisms for dealing with environmental stress and mental health issues (42). This finding is aligned with the results of a previous study (43).

According to the findings of previous studies (44, 45), there was an inverse association between depression symptoms and high economic status in the present study. The high prevalence of mental disorders in those with low economic status may be due to a number of factors, including not having enough income to meet even the most basic needs, the need for entertainment, the monotony of life, and a lack of hope for the future (40, 44, 45).

In line with other studies (10, 44, 45), the current study’s findings showed a positive association among depression and anxiety symptoms and a history of job loss, mental illness, feeling hopeless about the future, and having other medical conditions. The strongest association in this area was with feeling hopeless about the future; those who felt hopeless about the future were 5.38 and 3.33 times more likely to experience depression and anxiety symptoms, respectively. In the next stage, a history of mental disorders has the strongest association as the probability of depression and anxiety symptoms in people with a history of mental disorders was 2.17 and 2.11 higher, respectively than in those that did not have a history.

The present study does not show any association between age, body mass index, and marital status with depression symptoms. Although this finding is consistent with Mirzaei’s study (10), it is not consistent with other studies (40, 41, 44). Possible reasons for the inconsistency could be due to the use of different tools or different populations.

### 4.1. Limitations

Although the research team made great efforts to conduct the study perfectly, the present study also had limitations. Due to the cross-sectional nature of the study, the observed associations cannot be considered causal. We aimed to examine the condition of other mental disorders, but due to budget limitations, this was not possible. Finally, since the data was gathered with a self-report scale, the results of the present research are threatened with response bias. The high sample size, the use of a trained questioning team, and the selection of a representative random sample from the population of Ilam were the strong points of the present study.

### 4.2. Conclusion

The results of the study showed that hopelessness about the future, history of suffering from other diseases, history of mental disorders, job loss, low economic-social status, low education, ethnicity, and female sex are factors affecting mental disorders. Among these, hopelessness about the future and a history of other diseases were the strongest factors affecting mental disorders. Increasing people’s awareness, establishing counseling centers, especially telephone counseling, and improving recreational and sports facilities are very important, and mental health policymakers should pay special attention to them.

### 4.3. Implications for practice

The current study’s findings are applicable to local health policymakers through the reform and redesign of mental health programs in society. They should pay more attention to aspects that contribute to mental diseases, such as hopelessness about the future, by implementing innovative initiatives that can transform society’s attitude. Arranging happiness programs, spending more time on social media to orient social views into the future, developing infrastructure, and dealing with impoverishment are effective strategies for decreasing the prevalence of mental disorders. Furthermore, if researchers are aware of the considerable effect of hopelessness about the future on mental disorders, they might analyze this variable effect and which variables contribute to this association.

## Data availability statement

The raw data supporting the conclusions of this article will be made available by the authors, without undue reservation.

## Ethics statement

The studies involving human participants were reviewed and approved by This project has been approved by Ilam University of Medical Sciences with ethics code (IR.MEDILAM.REC.1401.031). Informed consent was obtained from all individual participants included in the study. Written informed consent to participate in this study was provided by the participants’ legal guardian/next of kin.

## Author contributions

RP, RV, and MM participated in the conception, design, data analysis, and wrote the manuscript. FM, HK, and AB contributed to the conception and design of the study and its review. HK and IP, the senior authors, were active in the conception, design, writing, and editing of the manuscript. All authors contributed to the article and approved the submitted version.

## Conflict of interest

The authors declare that the research was conducted in the absence of any commercial or financial relationships that could be construed as a potential conflict of interest.

## Publisher’s note

All claims expressed in this article are solely those of the authors and do not necessarily represent those of their affiliated organizations, or those of the publisher, the editors and the reviewers. Any product that may be evaluated in this article, or claim that may be made by its manufacturer, is not guaranteed or endorsed by the publisher.
